# Remarks on Strychnia as a Remedy in Chorea

**Published:** 1845-09

**Authors:** 


*Remarks on Strychnia as a Remedy in Chorea.*—In the same number of the *Lancet*, Dr. Ross, of Boulogne, calls the attention of the profession to strychnia as a remedy in chorea, and relates two cases in which it proved effectual; neither of these, from the description, however, appear to have been very aggravated forms of the disease.

The first case in which strychnia was employed by the author occurred in the year 1830; it was that of a delicate girl of 12 or 13 years of age, who came under his care as a hospital patient, having been previously for some weeks under other treatment. "Many of the eccentric symptoms of this singular disease were most distinctly marked; from having been very expert with her needle, she was rendered incapable of using it; her attempts to thread it were almost ludicrous." The strychnia was prescribed in the dose of the eighth or tenth of a grain twice a day; on the second or third day, through a mistake, an overdose was taken, which produced convulsive twitches, &c., but subsided on the discontinuance of the medicine, and with them all symptoms of the disease. In a day or two afterwards she was able to thread a fine needle, and at the end of a week she was dismissed cured. A year afterwards she had remained quite well.

The second case was a girl about the same age, also an hospital patient. The same treatment was adopted; no unpleasant effects followed the administration of the strychnia as in the former, and after a few days the symptoms gradually subsided, and she was dismissed well at the end of a fortnight.

Dr. Ross has not found the strychnia to bear out the favourable account which is given of it in the treatment of paralysis; but in chorea, in the limited number of cases in which he has employed it, he is much satisfied with its good effects, and recommends it for a further trial.—*Ibid.*

